# A Review on Biosensors and Recent Development of Nanostructured Materials-Enabled Biosensors

**DOI:** 10.3390/s21041109

**Published:** 2021-02-05

**Authors:** Varnakavi. Naresh, Nohyun Lee

**Affiliations:** School of Advanced Materials Engineering, Kookmin University, Seoul 02707, Korea

**Keywords:** biosensors, nanomaterials, nanobiosensing, gold nanoparticles, carbon nanotubes, quantum dots

## Abstract

A biosensor is an integrated receptor-transducer device, which can convert a biological response into an electrical signal. The design and development of biosensors have taken a center stage for researchers or scientists in the recent decade owing to the wide range of biosensor applications, such as health care and disease diagnosis, environmental monitoring, water and food quality monitoring, and drug delivery. The main challenges involved in the biosensor progress are (i) the efficient capturing of biorecognition signals and the transformation of these signals into electrochemical, electrical, optical, gravimetric, or acoustic signals (transduction process), (ii) enhancing transducer performance i.e., increasing sensitivity, shorter response time, reproducibility, and low detection limits even to detect individual molecules, and (iii) miniaturization of the biosensing devices using micro-and nano-fabrication technologies. Those challenges can be met through the integration of sensing technology with nanomaterials, which range from zero- to three-dimensional, possessing a high surface-to-volume ratio, good conductivities, shock-bearing abilities, and color tunability. Nanomaterials (NMs) employed in the fabrication and nanobiosensors include nanoparticles (NPs) (high stability and high carrier capacity), nanowires (NWs) and nanorods (NRs) (capable of high detection sensitivity), carbon nanotubes (CNTs) (large surface area, high electrical and thermal conductivity), and quantum dots (QDs) (color tunability). Furthermore, these nanomaterials can themselves act as transduction elements. This review summarizes the evolution of biosensors, the types of biosensors based on their receptors, transducers, and modern approaches employed in biosensors using nanomaterials such as NPs (e.g., noble metal NPs and metal oxide NPs), NWs, NRs, CNTs, QDs, and dendrimers and their recent advancement in biosensing technology with the expansion of nanotechnology.

## 1. Introduction

### 1.1. Sensors

Nowadays, we enjoy the results of science and technology for the smoothly running lives. We frequently rely on various types of appliances or gadgets, such as computers, copy machines, mobile phones, microwave ovens, refrigerators, air conditioning and television remotes, smoke detectors, infrared (IR) thermometers, turning on and off lamps and fans, which help us interact with the physical environment. Many of these applications perform with the help of sensors. A sensor is defined as a device or module that aids in detecting changes in physical quantities, such as pressure, heat, humidity, movement, force, and an electrical quantity like current, and thereby converts these to signals that can be detected and analyzed [1,2]. A transducer is defined as a device that can convert energy from one form to another. The sensor is the heart of a measurement system. An ideal sensor should possess certain characteristics, such as range, drift, calibration, sensitivity, selectivity, linearity, high resolution, reproducibility, repeatability, and response time [3,4]. The progress of sensor technology has become increasingly important, owing to various applications, such as environmental and food quality monitoring, medical diagnosis and health care, automotive and industrial manufacturing, as well as space, defense, and security.

### 1.2. Classification of Sensors

**Figure 1:**
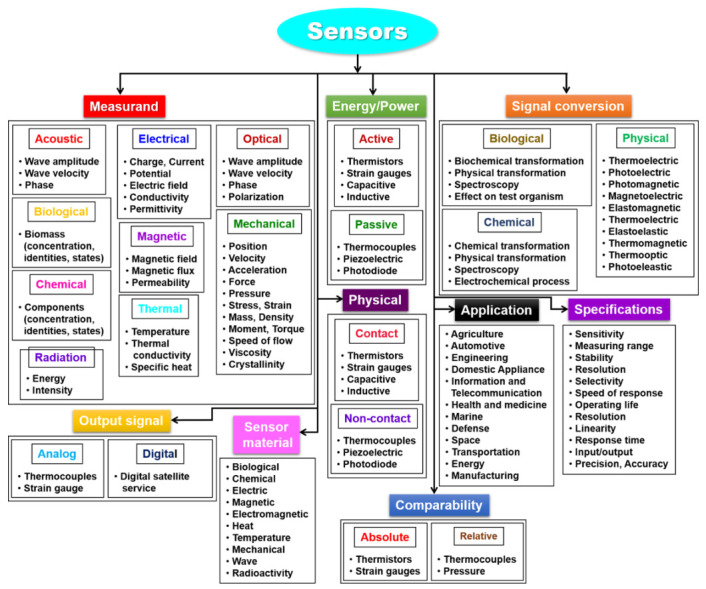
Classification of sensors based on measurand, energy/power, physical contact, signal conversion, output signal, comparability, sensor material, specification, and applications (reproduced from White et al. Ref. [6]).

Sensors are broadly classified into various categories (Figure 1) depending on the physical quantity (substance) or analyte to be measured, such as (a) energy source (active and passive sensors), (b) physical contact (contact and non-contact sensors), (c) comparability (absolute and relative sensors), (d) analog and digital sensors, and (e) signal detection (physical, chemical, thermal, and biological) [5,6]. Details of each classification are as follows.

(a) Active and passive sensors: Active sensors need an external energy source to operate, for example, microphones, thermistors, strain gauges, and capacitive and inductive sensors. These types of sensors are called parametric sensors (output is a function of the parameter). Passive sensors generate their signals but do not require external energy, for example, thermocouples, piezoelectric sensors, photodiodes. These types of sensors are called self-generating sensors.
(b) Contact and noncontact sensors: Contact sensors require physical contact with a stimulus, for example, temperature sensors, while non-contact sensors require no physical contact, such as optical and magnetic sensors and IR thermometers.
(c) Absolute and relative sensors: Absolute sensors, such as thermistor and strain gauge, react to a stimulus on an absolute scale. Relative sensors sense the stimulus relative to a fixed or variable reference, like a thermocouple that measures the temperature difference and the pressure that is measured relative to atmospheric pressure.
(d) Analog and digital sensor: An analog sensor transforms a measured physical quantity to an analog form (continuous in time). Thermocouples, resistance temperature detectors (RTD), and strain gauge belong to this class of analog sensors. A digital sensor generates output in the form of a pulse. Encoders belong to the digital sensor category.
(e) Signal detection: Based on the form of signal detection, sensors can be divided as (*i*) physical, (*ii*) chemical, (*iii*) thermal, and (*iv*) biological sensors.
  (*i*)  Physical sensors: Physical sensors measure a physical quantity and convert it into a signal, which can be identified by the user. These sensors can detect environmental changes, such as force, acceleration, rate of flow, mass, volume, density, and pressure. Physical sensors have been largely employed in the biomedical field, particularly with the advancement of microelectromechanical system (MEMS) technology for developing more precise and compact sensors, along with the development of novel measuring technology.
  (*ii*)  Chemical sensors: According to the international union of pure and applied chemistry (IUPAC), a chemical sensor is defined as, “a device that converts chemical information into an analytically useful signal ranging from the concentration of a particular sample component to total composition analysis.” Chemical sensor is employed to monitor the activity or concentration of the respective chemical species in the gas or liquid phase. They are also used for environmental pollution monitoring, food and drug analysis, and assay monitoring of organophosphorus compounds. They can also be used for clinical diagnostic purposes.
  (*iii*)  Thermal sensors: A thermal sensor is a device that is used to measure the temperature of an environment and transforms the input data into electronic data to record or monitor signal of temperature changes. Examples of temperature sensors include thermocouples, thermistors, and RTDs.
  (*iv*)  Biological sensors: Biological sensors monitor biomolecular processes, such as antibody/antigen interactions, DNA interactions, enzymatic interactions, or cellular communication processes. Biological sensors can be referred to as biosensors in short form.

## 2. Biosensor

### 2.1. Design and Principle

**Figure 2:**
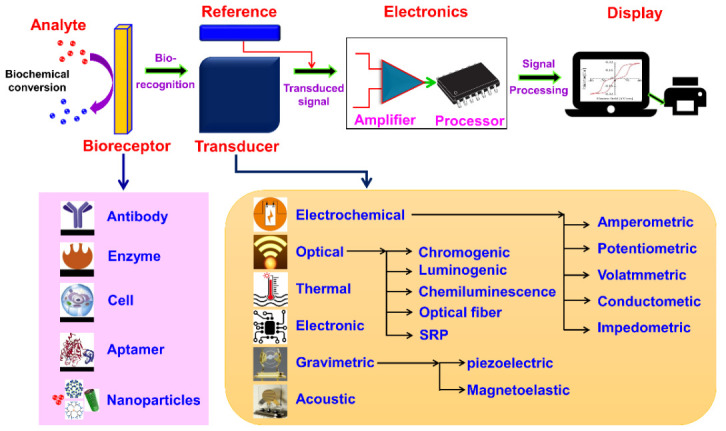
Schematic diagram of typical biosensor consisting of bioreceptor, transducer, electronic system (amplifier and processor), and display (PC or printer) and various types of bioreceptors and transducers used in the biosensors are also shown.

A biosensor is a device or probe that integrates a biological element, such as an enzyme or antibody, with an electronic component to generate a measurable signal. The electronic component detects, records, and transmits information regarding a physiological change or the presence of various chemical or biological materials in the environment. Biosensors come in different sizes and shapes and can detect and measure even low concentrations of specific pathogens, or toxic chemicals, and pH levels. A typical biosensor comprises (a) an analyte, (b) bioreceptor, (c) transducer, (d) electronics, and (e) display (Figure 2) [7].

(a) Analyte: A substance of interest whose constituents are being identified or detected (e.g., glucose, ammonia, alcohol, and lactose).
(b) Bioreceptor: A biomolecule (molecule) or a biological element that can recognize the target substrate (i.e., an analyte) is known as bioreceptor (e.g., enzymes, cells, aptamers, deoxyribonucleic acid (DNA or RNA), and antibodies). The process of signal production (in the form of light, heat, pH, charge or mass change, plant or animal tissue, and microbial products) during the interaction between bioreceptor and analyte is called biorecognition.
(c) Transducer: A device that transforms energy from one form to another. The transducer is a key element in a biosensor. It converts the biorecognition event into a measurable signal (electrical) that connects with the quantity or in the presence of a chemical or biological target. This process of energy conversion is known as signalization. Transducers generate either optical or electrical signals proportional to the number of analyte–bioreceptor interactions. According to the operating principle, transducers are broadly categorized as electrochemical, optical, thermal, electronic, and gravimetric transducers
(d) Electronics: The transduced signal is processed and prepared for the display. The electrical signals obtained from the transducer are amplified and converted into digital form. The processed signals are quantified by the display unit.
(e) Display: The display unit is composed of a user interpretation system, such as a computer or a printer that generates the output so that the corresponding response can be readable and understandable by the user. Depending on the end-user prerequisite, the output can be in the form of a numerical, graphical, or tabular value, or a figure.

### 2.2. Evolution of Biosensors

The evolution of biosensors has been classified into three generations based on the attachment of the components, that is, according to the method of integration of the bio-recognition element (bioreceptor) to the transducer. In the first generation (Ist gen), the biosensors measure the content of the analytes and products of the bioreceptor reactions, which diffuse to the surface of the transducer and produce an electric response. This type of sensor is also called mediator-less amperometric biosensors. Leland Charles Clark Jr., the father of biosensors, described components of a biosensor in his first report. This report, published in 1956, was about an electrode that can measure the oxygen concentration in blood [8]. In 1962, Clark experimentally described the employment of an amperometric enzyme electrode for glucose detection [9]. In 1967, Clark’s work was modified by Updike and Hicks, who realized the first functional enzyme electrode-based on glucose oxidase immobilized on an oxygen sensor [10].

**Figure 3:**
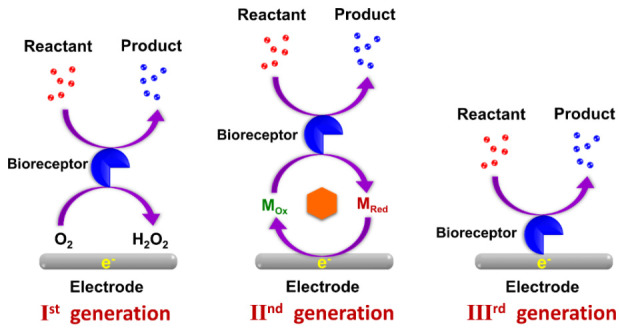
Three generations of the biosensor construction (M_OX_: Oxidized mediator; M_Red_: Reduced mediator).

In 1969, Guilbault and Montalvo demonstrated and reported the first potentiometric enzyme electrode-based sensor for detecting urea [11]. In 1973, Guilbault and Lubrano described glucose and a lactate enzyme sensor based on hydrogen peroxide detection at a platinum electrode [12]. A heat-sensitive enzyme sensor known as ”thermistor” was developed by the Klaus Mosbach group in 1974 [13]. In 1975, Lubbers and Opitz extended the concept to make an optical biosensor for alcohol [14]. In the second generation (IInd gen), individual components such as auxiliary enzymes and co-reactants (artificial or partially toxic mediators or nanomaterials), are integrated into the biological component layer of the biosensor with the view of enhancing analytical efficiency. These types of sensors are called mediator amperometric biosensors. In 1976, Clemens et al. incorporated an electrochemical glucose biosensor in a “bedside artificial pancreas” [15,16]. VIA Medical introduced a novel semi-continuous catheter-based blood glucose analyzer and, later in 1976, La Roche presented the lactate analyzer LA 640, which was used for electron transport from lactate dehydrogenase to an electrode [17]. In the third generation (IIIrd gen), the bioreceptor molecule becomes an integral part of the base sensing element, that is, biosensors progressed toward employing enzymes and mediators on the same electrode rather than freely diffusing mediators in the electrolyte. A direct interaction was established between the enzymes and electrode through the transfer of electrons, without any requirement of intermediate stages like in nanomaterials. Besides the interaction, low design cost and feasibility of having repeated measurements are the advantages of this generation of biosensor [18]. In 1983, Liedberg identified dependency reactions in real-time using the surface plasmon resonance (SPR) technique [19]. The blood glucose level was measured in 1987 with a pen-sized detector by Cambridge, USA. Figure 3 shows the three generations of the biosensors and Table 1 presents the timeline for the historical development of biosensors.

### 2.3. Characteristics of Biosensors

To develop a highly effective and capable biosensor system, certain static and dynamic requirements are necessary. Based on these specifications, the performance of the biosensors can be optimized for commercial uses [3,4,35].

(a) Selectivity: Selectivity is a crucial feature to consider when selecting a bioreceptor for a biosensor. A bioreceptor can detect a particular target analyte molecule in a sample comprised of admixture spices and unwanted contaminants.
(b) Sensitivity: The minimum amount of analyte that can be correctly detected/identified in a minimum number of steps and in low concentrations (ng/mL or fg/mL) to verify the existence of analyte traces in the sample.
(c) Linearity: Linearity contributes to the accuracy of the measured results. The higher the linearity (straight line), the higher the substrate concentration detection.
(d) Response time: The time is taken for obtaining 95% of the results.
(e) Reproducibility: Reproducibility is characterized by precision (similar output when the sample is measured more than once) and accuracy (capability of a sensor to generate a mean value closer to the actual value when the sample is measured every time). It is the ability of the biosensor to produce identical results whenever the same sample is measured more than once.
(f) Stability: Stability is one of the key characteristics in biosensor applications where continuous monitoring is required. Stability is the extent of vulnerability to environmental disturbances inside and outside the biosensing device. The factors that affect stability are the affinity of the bioreceptor (the extent of binding of the analyte to the bioreceptor) and the degradation of the bioreceptor over time.

### 2.4. Classification of Biosensors

**Figure 4:**
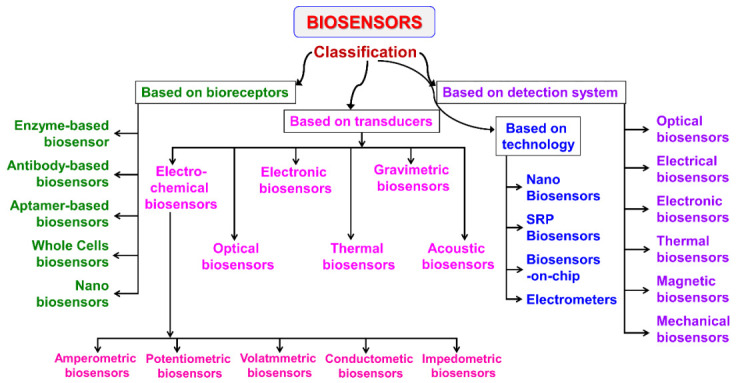
Classification of biosensors based on various bioreceptors and transducers used.

Classification of biosensors is a diverse and multidisciplinary field. Various criteria are involved in the classification of biosensors and the outline classification scheme is shown in Figure 4.

As discussed earlier, bioreceptors are considered as the primary component in biosensor construction. Based on the bioreceptor, biosensors are classified as enzymatic biosensors (most common biosensor class), immunosensors (possess high specificity and sensitivity and are specifically useful in diagnosis), aptamer or nucleic acid-based biosensors (possess high specificity for microbial strains and nucleic acid-containing analyte), and microbial or whole-cell biosensors. The second classification is made on the basis of the transducer and sensors are categorized as electrochemical (which is further grouped as potentiometric, amperometric, impedance and conductometric), electronic biosensor, thermal biosensor, optical, and mass-based or gravimetric. Another classification includes bioreceptor-analyte combinations, which are limited. Some classifications are made depending on the detection system (optical, electrical, electronic, thermal, mechanical, and magnetic) and the technology (nano, surface plasmon resonance (SPR), biosensors-on-chip (lab-on-chip), electrometers, and deployable).

### 2.5. Classification of Biosensors Based on Bioreceptors

According to the biorecognition principle, biosensors are classified as catalytic biosensors and affinity or non-catalytic biosensors [36]. In a catalytic biosensor, analyte–bioreceptor interaction results in the development of a new biochemical reaction product. This biosensor includes enzymes, microorganisms, tissues, and whole cells. In the case of affinity (non-catalytic) biosensor, the analyte is bound to the receptor irreversibly, and during the interaction no new biochemical reaction product is formed. This type of sensor comprises antibodies, cell receptors, and nucleic acids as the target for detection [37].

#### 2.5.1. Enzyme-Based Biosensors

Enzymes are common biocatalysts, which are efficient at increasing the biological reaction rate. The working principle of an enzyme-based biosensor depends on the catalytic reaction and binding capabilities for the target analyte detection [38]. Various possible mechanisms are involved in the analyte recognition process: (*i*) The analyte is metabolized by the enzyme, so the enzyme concentration is estimated by measuring the catalytic transformation of the analyte by the enzyme, (*ii*) an enzyme inhibited or activated by analyte, so the analyte concentration is related to decreased enzymatic product formation, and (*iii*) tracking of the alteration of enzyme characteristics [39,40,41,42]. Owing to the long history of enzyme-based biosensors, various biosensors can be produced on the basis of enzyme specificity. However, the enzyme structure is extremely sensitive, which makes it expensive and complicated to improve its sensitivity, stability, and adaptability [42]. Electrochemical transducers are most commonly used for enzyme-based biosensors. The most common enzyme-based biosensors are glucose and urea biosensors. Cordeiro et al. developed and characterized W-Au based amperometric enzyme-based glucose biosensors for real-time monitoring of glucose in the brain in vitro. Their experiments revealed that developed W-Au-based sensor can monitor changes in brain glucose in response to relevant pharmacological challenges [43]. Uygun et al. developed a highly stable potentiometric urea biosensor using nanoparticles. The response time and detection limit of their developed sensor were 30 s and 0.77 µM, respectively [44]. Integrating enzymes with nanomaterials has resulted in increased use of enzymes as recognition elements in biosensors [45].

#### 2.5.2. Antibody-Based Biosensors

Antibodies are affinity biorecognition elements, which have been used over two decades because of their wide application range and strong antigen–antibody interactions. Antibodies possess the structure of immunoglobulins (Ig) in the form of “Y” shape, which consists of two heavy and two light polypeptidic chains connected by disulfide bonds. Five classes of antibodies have been defined based on differences in heavy chains: IgG, IgM, IgA, IgD, and IgE [45]. Biosensors that have an embedded antibody as ligands or function on the antibody–antigen interaction are called immunosensors. Immunosensors are classified as (i) non-labeled and (ii) labeled. Non-labeled immunosensors are constructed to specifically determine the antigen–antibody complex by estimating the physical changes caused by the development of the complex. In the case of labeled immunosensor, a sensitively detectable label is introduced. The antigen–antibody complex is sensitively assessed through label measurement [46,47]. Madurro et al. constructed a label-free immunosensor to detect ovarium cancer. The system has a linear relationship of anti-CA125 concentration in the range of 5 to 80 U mL^−1^, exhibiting a limit of detection of 1.45 U mL^−1^ [48]. Marquette et al. developed electrical and optical biosensing for label-free detection of Aflatoxin B1 (AFB1) using gold (Au) nanobipyramids (NBPs). SPR-based AFB_1_ detection was found to be in the linear range of 0.1–500 nM with a detection limit of 0.4 nM, while impedimetric AFB_1_ detection was exhibited in the linear range of 0.1–25 nM, having a detection limit of 0.1 nM [49].

#### 2.5.3. Aptamer-Based Biosensors

Aptamers are synthetic single-stranded nucleic acids (sequences of DNA or RNA) that bind to target molecules selectively and can be folded into two-dimensional (2D) and three-dimensional (3D) structures. In 2D or 3D structures, the targets have high-binding performance due to greater surface density and less spatial blocking [50,51,52]. Due to the nucleic acid character of aptamers, they are structurally and functionally stable over a wide range of temperatures and storage conditions. Unlike antibodies that require biological systems to be generated, aptamers can be chemically synthesized, are stable in a pH range of 2–12, and have certain thermal refolding capabilities. A further benefit of aptamers is that they can be chemically modified according to the detection criteria for the target molecule [53]. By an in vitro selection mechanism, SELEX (Systematic Evolution of Ligands by EXponential enrichment), aptamers can be isolated from oligonucleotides libraries [54,55]. Several SELEX variants have recently been established, including cell-SELEX, capillary electrophoresis-based SELEX, microfluidics-SELEX, FACS-based SELEX, microtiter plate-SELEX, magnetic bead SELEX, and in vivo SELEX [56]. Optical, electrochemical, and piezoelectric techniques are the most frequently used in biosensors. Depending on different transduction techniques, these biosensors are further categorized as labeled or label-free aptasensors. Surface plasmon resonance (SPR) is the most commonly used method for the label-free optical sensors, whereas fluorescent dyes (fluorescein) are used for label-based optical aptasensors [57]. For tracking biological systems in real-time, fluorescent NPs, such as QDs, provides many benefits over regular fluorescent dyes. To identify targets, such as cancer cells, bacterial spores, and proteins, aptamer-QD conjugates were used [58,59]. Starno et al. demonstrated aptamer capped NIR PbS QDs to detect thrombin protein, based on selective charge transfer, within 1 min and with a detection limit of ~1 nM [60]. Gold nanoparticles (GNPs) have demonstrated interesting absorption characteristics that vary depending upon their aggregation state. Furthermore, GNPs are more biocompatible, easier to bioconjugate, and less toxic than QDs [61]. Feng et al. fabricated an electrochemical impedance spectroscopy (EIS)-based biosensor for L-Arg detection, which exhibited acceptable performance in the linear range of 0.1 pM–0.1 mM with a detection limit of 0.01 pM [62].

#### 2.5.4. Whole-Cell-Based Biosensors

Whole-cell-based biosensors utilize microbes (bacterial, fungi (yeasts and molds), algae, protozoa, and viruses), since they possess potential biorecognition elements, in the construction of whole-cell-based biosensors. They are self-replicating and can produce recognition elements, such as antibodies, without the need for extraction and purification [63,64]. Compared with animal or plant cells, whole-cell-based biosensors are easy to handle and rapidly proliferating. The cells can interact with a wide variety of analytes, display the electrochemical response that a transducer can register, and can transmit (whole-cell-based biosensor principle) [65]. Owing to their good sensitivity, high selectivity, and capability of detection, these biosensors were successfully employed in environmental monitoring, food analysis, pharmacology, heavy metals, pesticides, detection of organic contaminants, and drug screening [66]. Polizzi et al. developed label-free optical whole-cell *Escherichia coli* biosensors to detect pyrethroid insecticide exposure with a detection limit of 3 ng mL^−1^ 3-PBA in the linear range of 0.01–2 ng mL^−1^ [67].

#### 2.5.5. Nanoparticle-Based Biosensors

Apart from the above class of bioreceptors, NMs have recently been considered as a new category of bioreceptors. With the advancement of nanotechnology and nanoscience, different NMs have been used as bioreceptors [68]. NPs deliver a more vast range of applications in biosensing technology. NMs can act as bioreceptors as well as transducers. For example, cerium oxide-based NMs exhibit biomimetic catalytic activity favorable for bioreceptors [69]. Due to their effective transduction capabilities, different inorganic materials, such as graphene and CNTs-based NMs, noble metal NPs, and QDs, have successfully been used as transducers.

### 2.6. Immobilization Techniques in Biosensors

**Figure 5:**
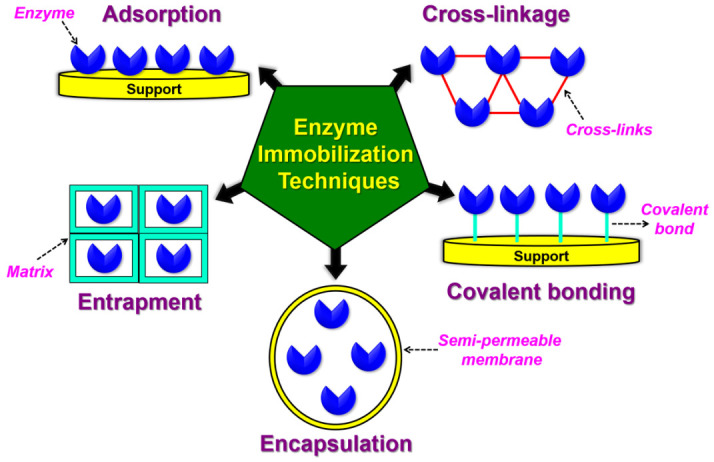
Enzyme immobilization techniques.

To design the biorecognition part of a biosensor, immobilization of biological elements on the surface of the transducer is necessary. One of the key aspects of sensor preparation is the selection of the appropriate immobilization technique. This is because after immobilization there will be chances for the biorecognition element/molecule to become inactivated or leached away. The biomolecules should be capable to preserve their structure, function, and biological activity during biosensor use. Enzyme immobilization techniques in biosensors are shown in Figure 5. Two important immobilization methods are widely familiar: Physical (reversible) and chemical (irreversible). The selection of a suitable technique relies on the biorecognition element chosen, the transducer, and the physical-chemical environment and on the characteristics of the analyte [61,70].

#### 2.6.1. Physical or Reversible Immobilization

This technique is based on attaching enzymes to the surface of the transducer without the creation of chemical bonds. These immobilizations include (a) physical adsorption, and (b) physical entrapment (electropolymerization, sol-gel technique, and microencapsulation) [36,70,71,72,73].

(a) Physical adsorption

In the adsorption technique, a biorecognition element immobilized on the outer surface of the inert solid material by weak attractive forces, such as van der waals force, the electrostatic force, ionic bonding, or hydrogen bonding. This method is seen in the case of enzyme biosensors. Advantages of physical adsorption method include its simple and economical process that it is non-destructive toward bioreceptor activity, and requires no modification of biological elements and generation of a matrix. The disadvantages of this method include that immobilization is susceptible to changes in pH, temperature, and ionic strength, is connected by a feeble interactions, and has poor operational and storage stability [61,70,71,72,73].

(b) Physical entrapment

In this process, biorecognition elements are physically entrapped within the 3D matrices via covalent or non-covalent bonds. The entrapment immobilization process can occur via two paths: The enzyme is mixed into a monomer solution, followed by polymerization of monomer solution by a chemical reaction, or by changing experimental conditions. The biorecognition elements are attached inside the 3D network of organic or inorganic materials. The organic materials used are polydimethylsiloxane, photopolymer, gelatin, alginate, cellulose, acetate phthalate, modified polypropylene, and polyacrylamide, whereas the inorganic materials are activated carbon and porous ceramic materials. Frequently used techniques in this process are (*i*) electropolymerization, (*ii*) the sol-gel process, and (*iii*) microencapsulation [61,70,71,72,73].

(*i*) Electropolymerization is a simple process in which a current or potential is applied to an aqueous solution or electrolyte containing both biomolecules and monomer molecules. Either reduction or oxidation of the monomer occurs on the surface of the electrode in the electrolyte, producing reactive radical species that join together and result in the formation of a polymer, which traps the biorecognition elements (enzymes) that are near the electrode in the aqueous solution. The electropolymerized films used for enzyme immobilization are aniline, pyrrole, and thiophene. [61,70,71,72,73].

(*ii*) The Sol-gel process is the most frequently used technique maintained at low-temperatures for enzyme trapping. A nanoporous material is formed via hydrolysis and condensation of metal alkoxides. As the network grows with time and temperature, a 3D matrix is formed in which the bio-elements are encapsulated. The advantages of this technique are thermal and chemical stability, easier way of synthesis, and the ability to encapsulate high concentrations of biomolecules at mild immobilizing conditions [61,70,71,72,73].

(*iii*) The Microencapsulation technique is a simple and inexpensive process where biorecognition elements (enzymes) are enclosed in a spherical semi-permeable membrane. The membrane can be of polymeric, lipoidal, lipoprotein based, or non-ionic. In microencapsulation, two methods are preferred: (i) Phase separation (coacervation) of enzyme micro-droplets in water-immiscible liquid phases and (ii) polymerization of a monomer at the interface of two immiscible substances (interfacial polymerization). This process results in the obscuration of the enzyme within the polymeric membrane [61,70,71,72,73].

#### 2.6.2. Chemical or Irreversible Immobilization

This method deals with the formation of strong chemical bonds, like covalent binding or covalent linking, between biorecognition element functional groups and the transducer surface. Based on the chemical bonding, chemical immobilization methods are categorized as (a) direct covalent binding and (b) covalent cross-linking [61,70,71,72,73].

(a) Covalent binding

Direct covalent binding is the most widely used enzyme immobilization technique, in which the biorecognition element is firmly bonded either to the electrode/transducer surface or to the inert matrix of the membrane. The immobilization process through the membrane matrix involves two steps: Synthesis of the functional polymer and covalent immobilization. The binding mechanism depends on the interaction between biorecognition element and functional protein groups (usually side chains of amino acids) and reactive groups of the transducer/membrane matrix surface. The advantages of direct covalent binding include strong resistance to environmental changes, little leakage of biorecognition element (enzyme), and strong bond formation between the biorecognition element (enzyme) and matrix. The main disadvantages of this method are the use of harsh chemicals and that the developed matrix cannot be regenerated once used [61,70,71,72,73].

(b) Cross-linking

The mechanism of cross-linking occurs via the creation of intermolecular covalent cross-linkages between biorecognition elements (enzymes) or between biorecognition elements and functionally inert proteins (for example bovine serum albumin). This process is performed with the help of multi-functional reagents that act as a linker to connect enzyme molecules in 3D cross-linked aggregates to the transducer surface. The optimal conditions required for cross-linking are pH, temperature, and ionic strength. It allows shorter response time, stronger attachment, and higher catalytic activity of enzymes. The advantages are less leakage of enzymes, stronger chemical binding, and the possibility to adjust the environment for the biorecognition element using appropriate stabilizing agents, The disadvantages are the formation of covalent cross-links between protein molecules instead of the matrix and protein and that partial denaturation of protein structure limit the application of cross-linking immobilization [61,70,71,72,73].

### 2.7. Classification Based on Transducers

According to their operating principle, transducers are broadly categorized as electrochemical, optical, thermal, electronic, and gravimetric (shown in Figure 4).

#### 2.7.1. Electrochemical Biosensors

**Figure 6:**
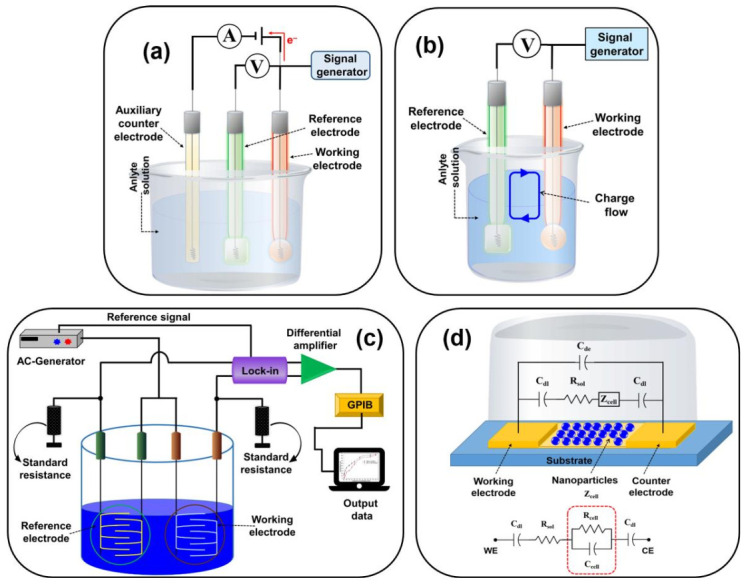
Schematic diagram of (**a**) amperometric/voltammetric, (**b**) potentiometric, (**c**) conductometric biosensors, and (**d**) equivalent circuit of the impedimetric biosensor (C_dl_ = double-layer capacitance of the electrodes, R_sol_ = resistance of the solution, C_de_ = capacitance of the electrode, Z_cell_ = impedance introduced by the bound nanoparticles, and R_cell_ and C_cell_ are the resistance and capacitance in parallel).

Electrochemical biosensors are the most widely investigated and used biosensors whose operating principles rely on the electrochemical properties of the analyte and transducer. Electrochemical biosensors exhibit high sensitivity, selectivity, and the capability of detection. In this biosensor, an electrochemical reaction occurs on the transducer surface between bioreceptor and analyte producing detectable electrochemical signals in terms of voltage, current, impedance, and capacitance [74,75]. Based on the transduction principle, electrochemical biosensors are categorized as: (a) Potentiometric, (b) amperometric, (c) impedimetric, (d) conductometric, and (e) voltammetric [74,75,76,77]. Figure 6 illustrates the schematic diagrams of (a) amperometric/voltammetric, (b) potentiometric, (c) conductometric, and (d) impedimetric (showing the equivalent circuit) biosensors.

(a) Potentiometric biosensors: Potentiometric biosensors measure the charge accumulated due to the analyte and bioreceptor interaction at the working electrode relative to the reference electrode under zero current. To transform a biochemical reaction into a potential signal, ion-selective electrodes and ion-sensitive field-effect transistors are used [74,75,76,77,78].
(b) Amperometric biosensors: Amperometric biosensors operate in a two or three-electrode configurations. These sensors measure the current produced due to electrochemical oxidation or reduction of electroactive species at the working electrode when a constant potential is applied to the working electrode with respect to the reference electrode. The current produced on the surface of the working electrode is proportional to the concentration of the analyte present in the solution [74,75,76,77]. Compared with potentiometric biosensors, this method allows sensitive, fast, precise, and linear response, which makes it more suitable for mass production. However, poor selectivity and interferences from other electroactive substances are the disadvantages of these sensors [79,80].
(c) Conductometric biosensors: Conductometric biosensors quantify the change in the conductance between the pair of electrodes because of an electrochemical reaction (change in conductivity properties of the analyte). Conductometric and impedimetric biosensors are usually used to monitor metabolic processes in living biological systems [76,81].
(d) Impedimetric biosensors: Impedimetric biosensors measure the electrical impedance produced at the electrode/electrolyte interface when a small sinusoidal excitation signal is applied. It involves the application of low amplitude AC voltage at the sensor electrode and then the in/out-of-phase current response is measured as a function of frequency using an impedance analyzer [76,82].
(e) Voltammetric biosensors: Voltammetric biosensors detect analyte by measuring the current during the controlled variation of the applied potential. Advantages of these sensors include highly sensitive measurements and simultaneous detection of multiple analytes [76].

#### 2.7.2. Optical Biosensors

**Figure 7:**
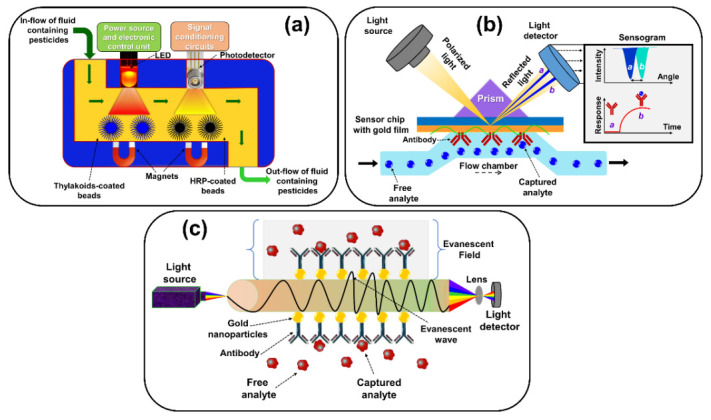
Schematic diagrams of (**a**) chemiluminescence biosensor, (**b**) surface plasmon resonance (SPR) biosensor, and (**c**) evanescent wave-based optical fiber biosensor.

Optical biosensors are analytical devices consisting of a biorecognition element integrated into an optical transducer system. The working principle of an optical biosensor is to generate signals, which are proportional to the concentration of the analyte and to provide label-free and real-time parallel detection. Optical biosensors utilize enzymes, antibodies, aptamers, whole cells, and tissues, as biorecognition elements. In optical biosensors, the transduction process induces a change in the absorption, transmission, reflection, refraction, phase, amplitude, frequency, and-/or light polarization, in response to physical or chemical changes created by the biorecognition elements. According to the principle, optical biosensors are categorized as label-free and label-based. In label-free sensing, the detected signal is produced by the interaction of the analyte with the transducer. On the contrary, in label-based sensing, the optical signal is generated by calorimetric, fluorescence, or luminescent methods. Optical biosensors can be designed based on various optical principles, such as SPR, evanescent wave (EW) fluorescence, optical waveguide interferometry, chemiluminescence, fluorescence, refractive index, and surfaced-enhanced Raman scattering. The most commonly used optical-based biosensors are (a) fluorescence-based optical biosensors, (b) chemiluminescence-based optical biosensors, (c) SPR-based optical biosensors, and (d) optical fiber-based optical biosensors [70,83,84,85]. In Figure 7, schematic diagrams of (a) chemiluminescence-based optical biosensors, (b) surface plasmon resonance (SPR)-based optical biosensors, and (c) evanescent wave-based optical fiber biosensors are shown.

(a) Fluorescence-based optical biosensors: Fluorescence is the optical phenomenon that includes labeling for the detecting analyte or molecules. This phenomenon has drawn much attention to the design of fluorescence-based optical biosensors. This type of biosensor is the most widely investigated for medical diagnosis, and environment and food quality monitoring applications because of its high selectivity, sensitivity, and short response time. Different kinds of fluorescent dyes, such as QDs, dyes, and fluorescent proteins, are used in this biosensor. Fluorescence-based biosensors include three approaches: (i) Fluorescent quenching (turn-off), (ii) fluorescent enhancement (turn–on), and (iii) fluorescence resonance energy transfer (FRET). Recently, fluorescence resonance energy transfer (FRET)-based optical biosensors have prevailed in the study if the intercellular process owing to their higher sensitivity. The FRET process involves nonradiative energy transfer from an excited donor molecule (D) to the acceptor molecule (A) at the ground state via long-range multipole interactions. As FRET-based optical sensors can detect changes in angstroms to nanometers, they are widely employed in clinical applications, such as cancer therapy and aptamers analysis [74,86]. Liu et al. constructed a carbon dots (CDs)/Au NR assembly-based FRET sensor for detection of lead ions. The linear range was obtained from 0 to 155μM with a detection limit of 0.05μM [87].
(b) Chemiluminescence-based optical biosensors: Chemiluminescence is the phenomenon in which light energy is released because of the chemical reaction. By virtue of its simplicity, low detection limit, wide calibration limit, and affordable instrumentation, chemiluminescence-based biosensors have received considerable interest. In recent times, chemiluminescence studies have been expanded to nanomaterials to improve intrinsic sensitivity and extended to the new applications of detection [88]. He et al. developed a chemiluminescence-based biosensor to detect DNA using graphene oxide, which exhibited high sensitivity and selectivity and the linear range is 0.1–3 nm with a limit of detection of 34 pM [89].
(c) SPR-based biosensors: SPR-based biosensors detect the change in the refractive index caused by molecular interaction at a metal surface through surface plasmon waves. This biosensor falls into the group of label-free biosensing technology and operates on the principle of SPR. According to the SPR phenomenon, when polarized light illuminates a metal surface at the interface between two media of different refractive indices, at a certain angle it produces electron charge density waves called plasmons. Based on the thickness of the layer at the metal surface, the SPR phenomenon results in the declined intensity of the reflected light relative to the incident light at a specific angle known as the resonance angle. The decrease in intensity is proportional to the mass on the surface [90,91]. Moreover, the SPR method relies on refractive index variations connected with the binding of the analyte to the biorecognition element on the transducer or SPR sensor. SPR phenomenon offers various applications in disease diagnosis and environmental and food quality monitoring. When the SPR phenomenon is extended to metal-based nanomaterials, such as gold and silver, NPs have given rise to a new phenomenon called localized surface plasmon resonance (LSPR). The main difference between LSPR and SPR phenomena is that plasma oscillations are governed by the total internal reflection locally at the surface of the nanostructure instead of at the metal surface [85,90,91]. The SPR-based DNA biosensor was developed by Rashidi et al. for detecting a donkey meat marker using gold nanostars. Its low detection limit of DNA was 1.0 nM with a relative standard deviation (RSD, *n* = 3) of 0.85% [92]. Hosseini et al. constructed a silver-based LSPR biosensor for the detection of endotoxin *E. coli.* with a detection limit of 340 pg mL^−1^ [93].
(d) Optical fiber-based optical biosensors: Optical fiber biosensor is an optical fiber-derived sensor system that employs an optical field to quantify biological species such as whole-cells, proteins, and aptamers. Optical fiber-based biosensors are considered as a promising alternative to the traditional methods employed for biomolecule assessment. The dependable optical fiber technique is an evanescent field sensing, which is observed in the case of tapered optical fibers. An evanescent wave is produced at the sample interface when light passes through an optical fiber as a result of total internal reflection. This field decays exponentially with distance from the interface. The evanescent wave can be used to excite fluorescence in the proximity of a sensing surface [94]. Tapered optical fibers are frequently used with different optical transduction processes, such as variation in refractive index, absorption, fluorescence and SPR [95,96]. Huang et al. developed a fiber optic sensor based on poly(N-isopropylacrylamide)-co-acrylamide)(P(NIPAAm-co-AAm))-magnetic immobilized glucose oxidase(GOD) complex (PMIGC) and glucose oxidase (GOD) to detect cholesterol and glucose. The sensor detected cholesterol (@ 38 °C) and glucose concentration (@ 25 °C) with the detection ranges of 25–250 mg dL^−1^ and 50–700 mg dL^−1^, respectively [97].

#### 2.7.3. Gravimetric Biosensors

**Figure 8:**
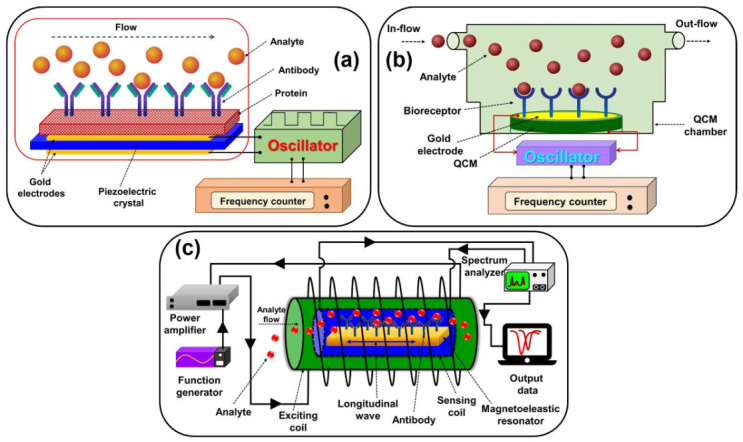
Schematic diagrams of (**a**) piezoelectric-based biosensor, (**b**) quartz crystal microbalance-based biosensor, and (**c**) magnetoelastic-based biosensor.

Gravimetric biosensors are mass-based biosensors, which respond to a small change in the mass of binding material, such as proteins or antibodies on the surface, producing a measurable signal. Gravimetric biosensors are employed with thin piezoelectric quartz crystals, which vibrate at a specific frequency according to the applied current and the mass of the detected material [98,99]. Figure 8 demonstrates schematic diagrams of (a) piezoelectric, (b) quartz crystal microbalance, and (c) magnetoelastic-based biosensors. Piezoelectric-based biosensors, magnetoelastic-based biosensors (MES), and quartz crystal microbalance (QCM) sensors are most commonly used for gravimetric transduction. These transducers are also used to identify pathogens and antigens by means of binding interactions. Piezoelectric biosensors are employed with a crystal that can undergo elastic deformation when a current or potential is applied. The alternating electric field at a specific frequency produces a wave in the crystal. If the analyte is absorbed or desorbed on the surface of the crystal, which is covered with the biorecognition element, the resonant frequency changes and suggests the occurrence of binding [100]. Yang et al. demonstrated a piezoelectric biosensor using a lead titanate zirconate (PZT) ceramic resonator as the transducer for label-free detection of cancer biomarkers. The developed device displayed a high sensitivity of 0.25 ng mL^−1^ in 30 min detection time for a small amount of sample (µL) [101]. QCM biosensors also work on the piezoelectric principle. A thin disk of quartz crystal is sandwiched between two conducting electrodes and the resonance frequency of crystal will change in response to the change in the mass of detected materials. Lee et al. developed sensitive and selective detection technology for miR-21 molecules using a QCM biosensor. The constructed QCM biosensor detected miR-21 with a detection limit of 0.87 pM in the linear range from 0.1 pM to 10 μM, with a correlation coefficient of 0.988. In addition to these results, this sensor was also highly effective in the measurement of miR-21 in serum samples, which can be an excellent alternative for clinical diagnosis [102]. MES has gained much attention as they are wireless and passive and can be used to determine force, stress, pressure, and strain. An MES comprises of thick-film-like amorphous ferromagnetic ribbons with high mechanical tensile strength (1000–1700 MPa). MES work on the principle of magnetostriction, in which mechanical deformation is developed as a result of the applied magnetic field. Magnetoelastic vibrations are produced when time-varying magnetic field is applied, causing the field-generated strain to change with time, which in turn produces longitudinal elastic waves. The elastic waves within the magnetoelastic material produce detectable magnetic flux. MES are ideal for biomedical applications owing to their cost effectiveness, long lifetime, small size, passive and wireless characteristics [103]. Atalay et al. used a magnetoelastic sensor to detect Fe_3_O_4_ magnetic nanoparticles. The minimum number of MNPs was measured to be about 1.1 × 109, which corresponds to 0.025mg or 1 µL of MNPs [104].

#### 2.7.4. Thermal Biosensors

**Figure 9:**
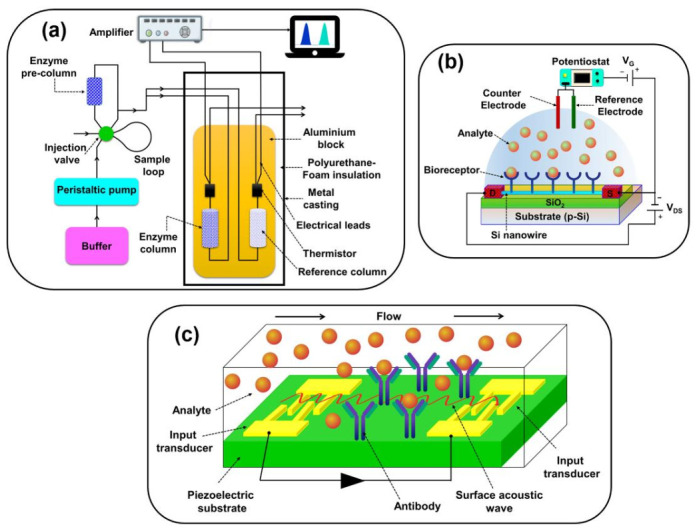
Schematic diagrams of (**a**) enzyme thermistor-based biosensor, (**b**) Si nanowire-based field-effect transistor (FET) (D is drain and S is the source) biosensor, and (**c**) surface acoustic wave (SAW)-based biosensor.

Thermal biosensor exploits the basic characteristics of the biological reactions (exothermic or endothermic), i.e., measurement of heat energy absorbed or released during the reaction. The total heat energy absorbed/evolved or the temperature change (Δ*T*) measured by thermal biosensor is proportional to the enthalpy (Δ*H*) and to the total number of product molecules (*n*_p_) created in the biochemical reaction and inversely proportional to heat capacity (*C*_p_) of the reaction, which can be shown as Δ*T* = −(*n*_p_ Δ*H*)/*C*_p_ [105,106,107,108,109]. Calorimetric-based biosensors measure the change in heat, which is directly monitored to calculate the extent of reaction (for catalyst) or structural dynamics of biomolecules in the dissolved state [108,110]. Calorimetric devices are limited because of the relatively long experimental procedures and lack of specificity in the temperature measurement; there is no direct way to discriminate between specific and non-specific heat changes. However, the invention of the enzyme thermistor-based on flow injection analysis in combination with an immobilized biocatalyst and heat-sensing element circumvented several of these shortcomings [107]. Usually, thermal biosensors employ a flow injection analysis method utilizing an immobilized enzyme reactor, together with a differential temperature measurement across the enzyme reactor. The configuration involves a pair of thermal transducers, such as thermistors or thermopiles, positioned across the enzyme column packed with immobilized enzymes for the conversion of given substrate to product. This differential measurement system generates a high common-mode-rejection ratio that greatly reduces the effects of ambient temperature fluctuations, allowing the specific measurement of enzyme catalysis. The thermal signals generated during the catalytic reaction sensed by the thermistor are proportional to the concentration of the substrate. Generally, the enthalpy changes for enzymatic catalysis are around −10 to −200 kJ mol^−1^, which is adequate to determine the substrate concentrations at clinically interesting levels for a range of metabolites [106,107,108,109,110]. Thermometric detection is advantageous when multiple reactions are involved since it is the sum of all enthalpies that determines the sensitivity of the assay. Thermistors or thermopiles are the two most commonly used temperature sensors. The thermistor is a sensitive temperature transducer, which depends on the changes in the electric resistance with temperature from which the absolute temperature can be determined but have limited sensitivity. Thermopiles measure the temperature difference between the two regions. Thermopiles are the set of thermocouple junctions in series fabricated from metals, semiconductors, and various substrate semiconductor components [108,109]. An enzyme thermistor-based biosensor is shown in Figure 9a. In addition to thermistors, bimetallic strips, liquid gas expansion, and pyroelectric systems (according to the production of an electrical signal because of a change in the temperature), metal resistance and microelectromechanical systems (MEMS) are being used as thermal transducers. Thermocouples with high sensitivity are also an excellent alternative for detecting changes in temperature [61]. Recently, microelectromechanical (MEMS) thermal sensors are being used to monitor metabolic applications on the basis of temperature detection. Low-cost integration of miniaturized devices and low-cost batch fabrication are the advantages of MEMS technology. MEMS thermal sensors exhibited improved thermal isolation, low thermal mass, and sample volume of the MEMS thermal biosensor provides linear range and high sensitivity, low measurement time, and low power consumption. It is possible to measure multiple samples in parallel [111].

#### 2.7.5. Electronic Biosensors

Figure 9b displays the Si NWs-based FET biosensors. The working principle of an electronic biosensor relies on field-effect transistors (FETs). The FET is a three-terminal device that regulates the current flowing through it using an electric field. They operate between the source and drain terminals on a semiconductor, whose impedance changes through the gate terminal [112,113,114]. Recently, FET-based transducers have gained much attention due to their capacity to directly translate the interactions between the analyte and FET surface [61]. If a biomolecule binds to the bioreceptor surface, it changes the surface potential. The corresponding change in channel width alters the current between the source and drain terminals [114]. The high input impedance of these semiconductor-based transducers is used to detect chemical changes from analyte and bioreceptor reactions. FET-based biosensors possess advantages compared to the other biosensing methods because of their high sensitivity and high spatial resolution, however they suffer some limitations when employed in vitro applications [115,116]. Frequently used transistor-based sensing platforms in biological applications are ion-sensitive field-effect transistors (ISFETs) and metal-oxide-semiconductor field-effect transistors (MOSFETs), depending on the technique of applying the gate voltage, design, and material of the gate and the channel region. Zhu et al. reported a ZnO NRs-based FET glucose sensor using AC frequency mixing detection rather than traditional three-electrode measurement in DC mode. The fabricated senor achieved a high sensitivity of 1.6 mA (µm-cm^2^)^−1^ with a detection limit of 1.0 µM glucose concentration. It also exhibited long-term stability of 38 h [117].

#### 2.7.6. Acoustic Biosensors

Acoustic biosensors operate based on the change in the physical properties of an acoustic wave in response that can be correlated to the amount of analyte absorbed [118]. Piezoelectric materials are commonly used for sensor transducers, because of their ability to produce and transmit acoustic waves in a frequency-dependent manner. For acoustic-wave propagation, the optimum resonant frequency is highly dependent on the physical dimensions and properties of the piezoelectric crystal. The changes in material mass on the surface of the crystal can induce measurable variations in the natural resonant frequency of crystal [100,101]. There are two classes of mass-balance acoustic transducers: Bulk-acoustic wave (BAW) and surface-acoustic wave (SAW) devices. BAW devices can transmit an acoustic wave from one crystal surface to another whereas SAW devices can transmit an acoustic wave along a single crystal face from one location to another [119,120]. The operation of these devices in the gas phase is well understood, but in liquid media, it is not that well understood. For many years the piezoelectric mechanism has been established and can be an alternative for the transduction process in affinity biosensors if the challenges of non-specific binding and poor sensitivity are solved [72,83]. The surface acoustic wave-based biosensor is shown in Figure 9c.

## 3. Advancement of Nanotechnology

**Figure 10:**
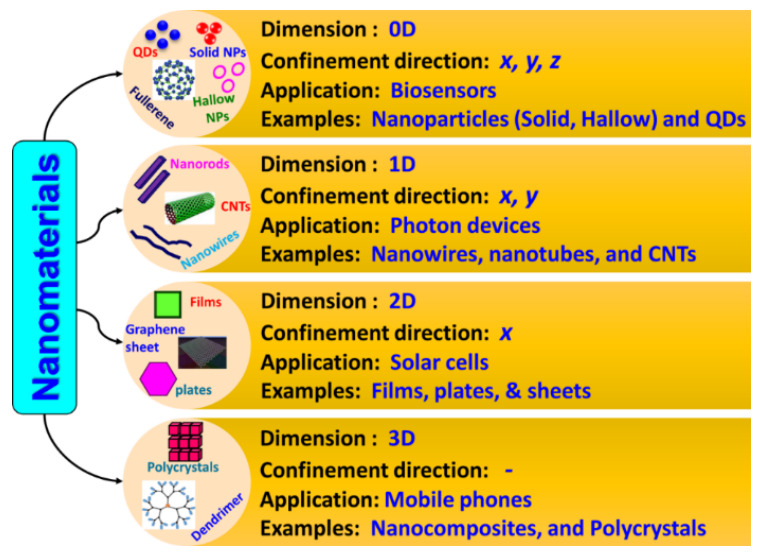
Classification of nanomaterials according to their dimensionality.

To meet the increasing demands of various fields, new approaches to sensor technology have been employed. Sensor technology has further flourished with progress in nanotechnology and nanoscience. Nanotechnology has stretched across various fields of science and industry, such as physics, chemistry, biotechnology, bioscience, bioinformatics, medical science, healthcare, food engineering or processing, aerospace, and electronics, the energy sector, and environmental studies. The ability to manipulate and control materials at an atomic and molecular level (nanometer range) and subsequent understanding of the fundamental processes at the nanoscale have led to new avenues of biosensor development. More importantly, dimensionality plays a major role in determining the characteristics of nanomaterials, including physical, chemical, biological, electrical, and optical characteristics. Nanomaterials are broadly classified, based on their nanoscopic dimensions, such as 0D, 1D, 2D, and 3D materials (Figure 10). If all three dimensions of a material are nano-sized, it would be called a 0D NM (NPs and QDs),). If two dimensions of a material are nanosized, with the other dimension being much larger, then it is 1D NM (NWs, NRs, NTs, nanobelts, and nanoribbons). If only one dimension is nanosized, it would be a 2D NM (nanoprisms, nanoplates, nanocoatings, nanolayers, nanosheets, nanowalls, nanodisks, and CNTs). Bulk nanomaterials are materials that are not confined to the nanoscale in any dimension (≥100 nm) and are referred as 3D NMs (nanoballs, dendritic structures, nanocoils, nanocones, nanopillars, multi-nanolayers, and nanoflowers) [121,122]. Synthesis of materials in the nanoscale range enables the unique physical, chemical, and biological properties and plays a pivotal role in the success of nanotechnology. Various approaches have been employed for the synthesis of NMs, categorized as a top-down approach (a bulk material is restructured to form nanosized materials) and a bottom-up approach (materials of nanodimension are formed by assembling molecule by molecule or atom by atom). The top-down approach includes various techniques, such as lithography, laser ablation, ion milling, and chemical etching. The commonly used techniques in the bottom-up approach are molecular beam epitaxy, physical or chemical vapor deposition and evaporation, and bio/chemical processes for the production of supra-molecular complexes, self-assembled monolayers, and protein-polymer nanocomposites [121,122].

## 4. Nanomaterial-Based Biosensors (Nanobiosensors)

**Figure 11:**
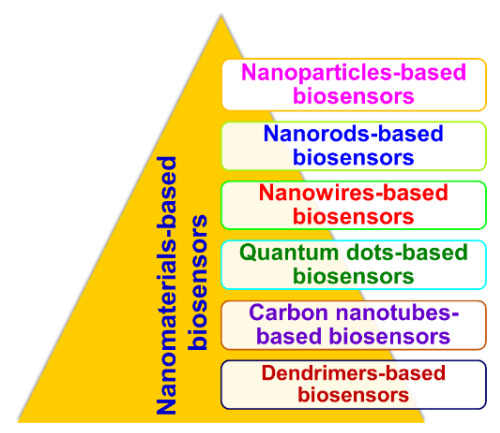
Types of nanomaterials-based biosensors (nanobiosensors).

With advances in nanotechnology, research and development in the field of biosensors have become open and multidisciplinary. Exploring NMs, such as NPs (metal- and oxide-based), NWs, NRs, CNTs, QDs, and nanocomposites (dendrimers), for different characteristics provides the possibility of improving the performance of biosensors and increase the power of detection through size and morphology control. Different types of NMs-based biosensors (nanobiosensors) are shown in Figure 11.

The basic working principle of nanobiosensors is along the same lines of conventional macro- and micro-counterparts, but they are constructed using nanoscale components for signal or data transformation [123]. Nanobiosensors have an edge over their conventional macro- and micro-counterparts because of their multidisciplinary applications due to dimensionality. Nanobiosensors are instrumental in the field of nanotechnology for (a) monitoring physical and chemical phenomena in regions difficult to reach, (b) detecting biochemicals in cellular organelles and medical diagnosis, (c) measuring nanoscopic particles in industrial areas and the environment, and (d) detecting ultra-low concentrations of potentially harmful substances [123]. Based on the classification of the NMs, their involvement in the enhancement of biosensing mechanisms has been broadly investigated. For instance, NPs-based biosensors include all sensors that employ metallic NPs as enhancers of biochemical signals. Similarly, nanotube-based biosensors, if they involve CNTs, are used as enhancers of reaction specificity and efficiency, while biosensors using NWs as charge transport and carriers are termed as NW biosensors. Likewise, QD-based sensors employ QDs as contrast agents for improving optical responses.

### 4.1. Nanoparticle-Based Biosensors

NPs have been widely used in various biomedical applications, like in the development of biosensors for health diagnosis, imaging, drug delivery, and therapy, owing to their unique properties. Because of their small size and shape, their physical and chemical properties are strongly influenced by the binding of target biomolecules [124]. These properties of NPs enable them to be exploited for various bioanalytical applications. They are considered suitable for electrode modification in which they increase the sensitivity and specificity of electrochemical catalysis [70]. Moreover, catalytic active NMs, such as transition metal oxides, have been developed as nanoenzymes, which allow providing catalysis of biochemical reactions on biosensors. The NPs include metal and noble metal NPs, such as gold (Au), silver (Ag), platinum (Pt), palladium (Pd), cobalt (Co), iron (Fe), and copper (Cu), and metal oxide NPs (ZnO, TiO_2_, SnO_2_ and MnO_2_), which exhibit excellent optical, electronic, magnetic, chemical, mechanical, and catalytic properties. NP biosensing performance is tailored by coating with various matrices, such as metal oxides, silica network, polymers, graphene, fibers, and dendrimers [125].

Gold NPs, which fall into the class of noble metal NPs, are extensively investigated and used owing to their unique optical, electronic, and physicochemical properties. They are widely used in biomedical research because of the following advantages: Simple synthesis techniques, easier fabrication procedures, greater chemical stability, biocompatibility, vast electrochemical potential range, high catalytic activity, and their nanocomposite forms [126,127]. Wu et al. demonstrated gold NP-based electrochemical sensors for sensitive detection of uranyl in natural water. The developed sensor determined uranyl in the range of 2.4 to 480 µg L^−1^, and a detection limit of 0.3 µg L^−1^ was obtained by anodic stripping voltammetry [128]. Luo et al. established a novel “turn–on” fluorescent sensor for detecting Pb^2+^, based on graphene quantum dots (GQDs) and gold nanoparticles (AuNPs). The designed sensor showed an extremely broad detection range of Pb^2+^ from 50 nm to 4 µm, with a detection limit of 16.7 nm [129]. Ghasemi et al. demonstrated a novel non-enzymatic glucose sensor based on gold-nickel bimetallic NPs doped aluminosilicate framework prepared from agro-waste material that exhibited a wide linear range for glucose (1–1900 µM) and low limit of detection (0.063 µM) [130].

Silver nanoparticles (Ag NPs) have gained much research interest in biomedical applications due to excellent surface-enhanced Raman scattering (SERS), biocompatibility, high conductivity, amplified electrochemical signals, and catalytic activity [131,132,133]. Rivero et al. demonstrated an optical fiber sensor based on both localized surface plasmon resonance (LSPR) and lossy-mode resonance (LMR) using Ag NPs. The devices showed high sensitivity (0.943 nm per RH %), a large dynamic range (42.4 nm for RH changes between 25% and 70%), and a fast response time (476 ms and 447 ms for rise and fall, respectively). This device can be used to monitor human breathing [134]. Mehdinia et al. developed a multi-functional colorimetric probe for Fe^2+^, H_2_O_2_, and glucose detection based on the Fenton reaction and biosynthesized AgNPs. The low detection limit for Fe^2+^ was 0.54 µM, for H_2_O_2_ was 0.032 µM, and for glucose was 0.29 µM [135].

Over the past decade, owing to their unique electronic and electro-catalytic characteristics, platinum nanoparticles (Pt NPs) have gained much interest in the field of electrochemical biosensors for biomedical applications. The electron transfer process in Pt NPs is affected by material composition, surface reactive environment, crystalline plane, and orientation [133]. Liu et al. fabricated a new Pt NP/a-IGZO-based ammonia sensor showing high sensing response (*S*_R_) of 1467 (at 1000 ppm NH_3_/air, 250 °C and exhibiting fast sensing speed [136]. Dharuman et al. developed a graphitic carbon nitride modified with Pt and zinc oxide NPs for non-enzymatic glucose sensing with a wide linear detection range of 250 μM to 110 mM. It can be reusable four times in whole blood and eight times in blood serum [137].

Palladium nanoparticles (Pd NPs) are another fascinating NP for biomedical applications because of their high catalytic and sensing activities. In addition, palladium (Pd) is much more abundant than Au and Pt metals making it employable in various sensors for sensing applications in a cost-effective way. Pd NPs with controllable size and shape exhibit high electro-catalytic and sensing characteristics for different chemical and biological analytes [135,138]. Ye et al. prepared a Pd/Co-NCNTs exhibited excellent electrocatalytic ability for hydrazine oxidation and showed a high sensitivity of 343.909 μA mM^−1^ with a low detection limit of 0.007 μM for hydrazine [139]. Swihart et al. developed a unique 3D Pd-decorated crumple reduced graphene oxide ball (Pd-CGB) nanocomposite for hydrogen (H_2_) detection in air at room temperature. The sensitivity of the sensor is measured for the H_2_ concentrations (0.0025–2%) with response value, response time, and recovery time of 14.8%, 73 s, and 126 s, respectively, at 2% H_2_ [140]. Afzali et al. developed a novel sensor based on Pd/CNF/[M3OA]^+^[NTF2]^−^ modified CPE through a sensitive square-wave voltammetric procedure for the determination of the anticancer drug pemetrexed. A linear concentration was detected in the range of 1.00–35.0 nM with a detection limit of 0.33 nM by square wave voltammetry (SWV) technique [141].

Copper (Cu) has gathered much research interest as a unique sensing material owing to its excellent electrical conductivity, stability, electrocatalytic properties, and low cost compared to noble metals. Recently, Huang et al. prepared and studied the performance of electrochemical glucose sensors based on copper nanoparticles (Cu NPs) loaded on a flexible graphite sheet. The developed sensor exhibited a low detection limit of 1.05 µmol L^−1^ and high sensitivity of 7254.1 μA mM^−1^ cm^−2^, with R^2^ = 0.9961 from 0.1 to 3.4 mmol L^−1^ and 3804.5 μA mM^−1^ cm^−2^ (R^2^ = 0.9995) from 3.4 to 5.6 mmol L^−1^. Cu NPs also exhibited excellent anti-interference properties against sodium chloride, acetaminophenol, ascorbic acid, dopamine, and uric acid, with good reproducibility [142]. Roushani et al. developed a novel sensor for analytical detection of H_2_O_2_ based on the incorporation of CuNPs onto an MWCNTs/IL/Chit/Rutin nanocomposite film. The electrochemical performance of the sensor for detecting H_2_O_2_ was investigated by cyclic voltammetry and chronoamperometry techniques. The response to H_2_O_2_ was linear, in the range of 0.35 μM to 2500 μM, with a detection limit of 0.11 μM [143]. Zhao et al. fabricated a Cu/rGO decorated buckypaper electrode for glucose detection. The constructed device exhibited a linear range of 0.1–2 mM, with a detection limit of 11 µM [144].

#### Metal Oxide-Based Nanoparticles

Over the last decade, metal oxide-based nanomaterials have been vividly employed in various fields, such as electrochemistry, magnetism, catalysis, and sensors, because of their broad range of electrical, chemical, and physical properties. These oxide-based materials are used as an effective electrocatalyst for sensing various analytes in the field of biology and biomedicine because of their strong electrocatalytic activity, low cost, and high organic capture ability. The most often employed metal oxide nanoparticles include copper oxide (CuO), nickel oxide (NiO), iron oxide (Fe_2_O_3_), cobalt oxide (Co_3_O_4_), manganese oxide (MnO_2_), zinc oxide (ZnO), titanium oxide (TiO_2_), tin oxide (SnO_2_), cadmium oxide (CdO), molybdenum oxide (MoO_3_), and cerium oxide (CeO_2_) [145].

Nickel oxide-based nanoparticles (NiO NPs) exhibit superior electrical, magnetic, optical, thermal, catalytic, and mechanical properties. NiO-based nanostructures have been used as catalysts, thermistors, sensors, and additives, for gases and ceramics. NiO NPs are also p-type semiconductors with a direct bandgap of (3.56 eV) that can exhibit super-paramagnetic, as well as superanti-ferromagnetic properties, depending on their size and oxidation states [146]. Recently, Recently, Duan et al. fabricated high performance FET glucose biosensors based on bimetallic Ni/Cu metal organic frame works. The fabricated device exhibited a low detection limit of 0.51 µM with a linear range of 1 µM–20 mM [147]. Kamyabi et al. fabricated novel electroluminescence (ECL) glucose biosensors based on immobilized glucose oxidase in the cavity of nickel foam modified with NiO NPs. The proposed ECL biosensor showed superior performance toward glucose in 0.1 M phosphate buffer solution (pH 7.4) with a wide linear range (2.7 × 10^−9^ to 4.5 × 10^−3^ M) and a low detection limit (5.0 × 10^−10^ M) [148].

Cobalt oxide-based nanoparticles (Co_3_O_4_ NPS) have attracted considerable interest due to their exceptional physical, magnetic, optical, electronic, and chemical properties. They possess promising applications, such as catalysts, solar selective absorber, gas sensors, lithium-ion batteries, supercapacitors, and pigment for glasses, and ceramics, photocatalysis, magnetic material, and chemical sensors. It is a p-type semiconductor with a direct optical band gap of 1.66 and 2.19 eV [149]. Chu et al. developed a screen-printed glutamate biosensor chip using porous Co_3_O_4_ nanocubic crystals. The developed biosensor chip exhibited a high sensitivity of 20.12 μA mM^−1^ cm^−2^, as well as a wide linear range from 10 to 600 μM and a detection limit of 10 μM [150]. Wazir et al. developed a potentiometric urea biosensor fabricated on glass filter paper through the immobilization of urease enzyme onto cobalt oxide- chitosan nanocomposite. The sensitivity was measured over the concentration range between 1 × 10^−4^ and 8 × 10^−2^ M of the urea electrolyte solution, revealing that the fabricated biosensor exhibited good sensing ability with a linear slope curve of 45 mV/decade [151]. Ge et al. constructed a Co_3_O_4_-Au polyhedron-based photoelectrochemical (PEC) biosensor for detecting miRNA-141 detection with a linear range of 1 pM to 50 nM, and a detection limit of 0.2 pM [152].

Iron oxide (Fe_2_O_3_) and manganese oxide (MnO_2_)-based NPs are considered the best known magnetic NMs because of their bioanalytical applications and higher electron transfer rates. They are also considered to be promising materials for electrochemical biosensors [153,154]. Phan et al. demonstrated the possibility of using the magneto-reactance effect of a soft ferromagnetic amorphous ribbon with a nanohole-patterned surface to develop a highly sensitive magnetic biosensor for detection and quantification of anticancer drugs tagged to super-paramagnetic NPs [155]. Zhang et al. developed a fast and highly specific LF-NMR biosensor that can directly detect *Salmonella*, without sample pretreatment, with a detection limit of 2.6 × 10^4^ CFU mL^−1^ [156]. Stankovic et al. developed a disposable biosensor based on graphene nanoribbons supported with MnO_2_ NPs. The sensor displayed a detection limit of 0.05 mmol L^−1^ and a high sensitivity of 56.32 μA mmol^−1^cm^−2^ [157].

Other metal oxide-based NPs, such as ZnO, TiO_2_, SnO_2_, and MoO_3_, have recently gained much attention. ZnO based nanoparticles possess good electron transfer rate and thermal/chemical stability, oxidation resistance, biocompatibility, and high conductivity. ZnO is an n-type semiconductor with a wide bandgap energy of 3.37 eV [158,159]. Hjiri et al. prepared a carbon monoxide sensor using ZnO NPs synthesized by the sol-gel technique. The developed gas sensor exhibited a response of 74% toward 80 ppm of CO gas with a response/recovery time of 21 and 70 s, respectively, at 250 °C and high stability with time [160]. SnO_2_-based NPs have also been used for detecting toxic gases and pesticide sensing applications [161,162]. TiO_2_-based NPs can be used in electrochemical sensors for medical, biomedical, and pharmaceutical applications. Tereshchenko et al. introduced a novel and simple optical immunosensor to determine *Salmonella typhimurium* based on TiO_2_ NPs deposited on a glass substrate with a sensitivity in the range of 10^3^–10^5^ cL mL^−1^ [163]. Recently, Ravikumar et al. reported on rapid and facile method for detecting H_2_O_2_ in chemical reactions using molybdenum oxide (MoO_3_) NPs [164].

### 4.2. Quantum Dot-Based Biosensors

QDs are inorganic nanocrystals (NCs), which belong to 0D NMs displaying unique optical properties of broad excitation, narrow size-tunable emission spectra, high photochemical stability, and negligible photo-bleaching [165]. They have been widely used, mainly as alternatives to fluorophores, for developing optical biosensors to detect organic compounds, pharmaceutical analytes, and biomolecules, such as nucleic acids, proteins, amino acids, enzymes, carbohydrates, and neurotransmitters [166]. They have also been employed for the in vivo detection of cancer. They are ideal candidates for multiplexed optical bioanalysis due to their ultra-high sensitivity, high specificity, cost-effectiveness, miniaturized size, size-dependent emission wavelength, and rapid analyte detection [167]. Cui et al. proposed a simple and efficient electrochemical sensor for Cu (II) based on GQDs and graphene. The combination of GQDs and graphene-enhanced the performance, with a low detection limit of 1.34 nM in a wide linear range of 0.015 μM to 8.775 μM for Cu (II) [168]. Packirisamy et al. demonstrated the fabrication and application of fluorescent turn-on biosensors for ultrasensitive detection of small cell lung cancer biomarkers using biofunctionalized GQDs as the energy donor and AuNPs as energy acceptor. The fluorescent biosensor exhibited a fast response time (16 min), and broader linear detection range (0.1 pg mL^−1^ to 1000 ng mL^−1^), and low detection limit of 0.09 pg mL^−1^ [169]. Xiao et al. demonstrated CdTe/CdS/ZnS core/shell/shell QDs-based fluorescent biosensors for the determination of L-ascorbic acid. The concentration was detected in the linear range of 8.0 × 10^−9^ to 1.0 × 10^−7^ M with a detection limit of 1.8 × 10^−9^ M [170]. Sun et al. described a “turn-on” magnetic fluorescent biosensor based on GQDs, Fe_3_O_4_, and molybdenum disulfide (MoS_2_) nanosheets for detecting EpCAM in the linear range between 2 and 64 nM with a detection limit of 1.19 nM. It is used for rapid, efficient, and sensitive separation and detection of circulating tumor cells (CTCs) [171].

### 4.3. Nanowire-Based Biosensors

NWs provide favorable conditions for creating robust, sensitive, and selective electrical detectors of biological binding events. The NWs exhibit highly reproducible optical and electrical characteristics. Current flow in any 1D systems, such as NWs and NTs is extremely sensitive to minor perturbations because, in such systems, flow is extremely close to the surface [172]. The diameter of the NWs are comparable to the biological and chemical species that are being sensed. They offer excellent transduction generating signals, which ultimately interface to macroscopic instruments. The combination of tunable conducting properties of semiconducting NWs and the ability to bind analytes on their surface yields a direct, label-free electrical readout [173]. NWs-based sensors operate on the principle of ion-selective FETs and rely on the interaction of external charges with carriers in the nearby semiconductor, which results in enhanced sensitivity at low ionic strength. Park et al. fabricated fiber optic sensor using ZnO NWs and AuNPs for highly sensitive plasmonic biosensing [174]. Priolo et al. demonstrated label-free and PCR-free silicon NWs-based optical biosensor for direct genome detection. They exhibited a detection limit of 2 copies per reaction for the synthetic genome and 20 copies per reaction for the genome extracted from human blood [175]. Nuzaihan et al. prepared a silicon NW-based biosensor with novel molecular gate control for electrical detection of Dengue virus (DENV) DNA. The developed sensor had a low detection limit of 2.0 fM concentration with high sensitivity of 45.0 μA M^−1^ [176].

### 4.4. Nanorods-Based Biosensors

Nanorods are often used as simple electrochemical modifiers providing a highly specific process. They are usually prepared from gold, graphene, manganese, zinc, or iron oxide, or the combination of these materials [177,178]. The detection of nucleic acid or basic biochemical markers, such as glucose and hydrogen peroxide, are their most common applications. Liu et al. constructed a new CDs/Au NR assembly-based FRET sensor for detecting lead ions. A linear range from 0 to 155 μM, with a detection limit of 0.05 μM [87]. Zhu et al. demonstrated a ZnO NRs-based FET biosensor for continuous glucose monitoring using AC frequency mixing. The fabricated sensor achieved a high sensitivity of 1.6 mA (μM^−1^ cm^−2^) with a concentration detection limit of 1 μM, and exhibited an excellent long-term stability on continuous monitoring for 38 h [117]. Sun et al. used GNRs and graphene oxide (GO) to enhance the sensitivity of a wavelength modulation SPR biosensor to detect bovine IgG. The developed biosensor based on GNRs/GO sensing had detected bovine IgG in the concentration range of 0.075–40.0 µg m L^−1^ [179]. Hahn et al. fabricated a vertically grown ZnO NRs-based FET biosensor to detect phosphate with high sensitivity (80.57 μA mM^−1^ cm^−2^) in a wide linear range (0.1 µM–7.0 mM) [180].

### 4.5. Carbon Nanotubes-Based Biosensors

CNTs are exciting 1D NMs and are the most extensively investigated nanotubes class of materials in biosensors, diagnostics, tissue engineering, cell tracking and labeling, drug delivery, and biomolecules. They are hollow cylindrical tubes composed of one, two, or several concentric graphite layers capped by fullerenic hemispheres, which are referred to as single-, double-, and multi-walled CNTs, respectively. They have unique structures, excellent electrical and mechanical properties, high thermal conductivity, high chemical stability, remarkable electrocatalytic activity, minimal surface fouling, low over-voltage, and high aspect ratio (surface-to-volume) [181,182,183]. Because of their high surface-to-volume ratio and novel electron transport properties, the electronic conductance of theses nanostructures is strongly influenced by minor surface perturbations, such as those associated with the binding of macromolecules. CNT-based biosensors and diagnostics have been employed for the highly sensitive detection of analytes in healthcare, industries, environmental monitoring, and food quality analysis. They have been predominantly used in electrochemical sensing, for glucose monitoring, but also for detecting fructose, galactose, neurotransmitters, neurochemicals, amino acids, immunoglobulin, albumin, streptavidin, insulin, human chorionic gonadotropin, C-reactive protein, cancer biomarkers, cells, microorganisms, DNA, and other biomolecules. Multi-wall-carbon nanotubes (MWCNTs) are represented in all applications of nanotubes in biosensing. Such 1D nanomaterials provide real-time and sensitive label-free bioelectronic detection and massive redundancy in nanosensor arrays [165]. Cui et al. developed a wearable-based amperometric biosensor painted onto gloves as a new sensing platform used to determine lactate [184]. Janssen et al. demonstrated a CNT-based biosensor to sense a standard protein, bovine serum albumin (BSA), as a proof-of-concept. The developed sensor had a detection limit of 2.89 ng mL^−1^ [185]. Tang et al. fabricated a single-walled carbon nanotube (SWNT)-based DNA sensors and described the sensing mechanism. This work demonstrated clear experimental evidence on SWNT-DNA binding on DNA functionality, which paved a path for the future design of SWNT biocomplexes for applications in biotechnology and DNA-based nanotube manipulation techniques [186]. Hong et al. constructed metallic floating electrode-based DNA sensors with controllable responses. They showed the enhancement in the sensor response on increasing the number of floating electrodes [187]. Park et al. demonstrated a CNT-based biosensor system-on-a-chip for detecting a neurotransmitter. Here, CNT-based sensors were integrated with CMOS chips, which is useful in various biomedical applications, such as sensing components in LoC (lab-on-a-chip) systems for neuronal culture [188].

### 4.6. Dendrimer-Based Biosensors

Dendrimers are nanometer-scale 3D macromolecules in the size of an average protein-, and are hyper-branched, mono-dispersed, and star-shaped, with a high density of surface functional groups. The shape of dendrimers provides a vast surface area for the conjugation of biologically active molecules. They are composed of three distinct components: The core, the interior dendron, and the exterior surface with terminal functional groups [189,190]. They have been used extensively in various biosensors, diagnostics, and drug delivery based on electrochemistry, fluorescence, SERC, impedimentary, and SPR. Dendrimer-based biosensors increase analytical sensitivity, stability, and reproducibility but reduce no specific interactions [191,192,193]. Bakar et al. detected dengue using a PAMAM dendrimer integrated tapered optical fiber sensor. The resolution and detection limit of the sensor were 19.53 nM^−1^ and 1 pM, respectively, in the concentration range of 0.1 pM to 1 µM [194]. Fen et al. developed an SPR sensor based on self-assembled monolayer/reduced graphene oxide-polyamidoamine dendrimer (SAM/NH_2_rGO/PAMAM) thin films to detect DENV-2 E-proteins. Their SPR sensor exhibited a detection limit of 0.08 pM DENV-2E-proteins in the range of 0.08 pM–0.5 pM [195]. Table 2 represents the list of various nanomaterials employed in the development of biosensors.

## 5. Conclusions

In this review article, we have discussed types and mechanisms of biosensors based on receptors (enzymes, antibodies, whole-cell, and aptamers), transducers (electrochemical, electronic, optical, gravimetric, and acoustic), and nanomaterials (gold NPs, Ag NPs, Pt NPs, Pd NPs, NWs, NRs, CNTs, QDs, and dendrimers). Biosensors offer versatile applications in the fields of engineering and technology, medicine and biomedical, toxicology and ecotoxicology, food safety monitoring, drug delivery, and disease progression. With the application of NMs in biosensors, we have witnessed rapid growth in biosensing technology is witnessed in the recent decade. This is because of the employment of new biorecognition elements and transducers, progress in miniaturization, design, and manufacture of nanostructured devices at the micro-level, and new synthesis techniques of NMs, all of which bring together the life and physical scientists and engineering and technology. The sensing technology has become more versatile, robust, and dynamic with the induction of nanomaterials. The transduction mechanism has been improved significantly (like greater sensitivity, faster detection, shorter response time, and reproducibility) by using different nanomaterials (such as NPs, NRs, NWs, CNTs, QDs, and dendrimers) that each has different characteristics within biosensors. Though there is considerable improvement in the use of nanostructured materials in biosensors applications, there are few limitations, which hinder these applications for the next level. For instance, lack of selectivity remains a setback for the CNT-based gas sensors, hampering its usage in CNT-based devices. However, this hurdle can be overcome by coupling CNTs with other materials. The other issues in these sensors include (i) the sustainability of nanostructures in sensor applications, which have been insufficiently investigated, (ii) the fabrication of nanostructures, and (iii) the toxicity, which changes according to the physical properties of the material type. These issues should be investigated and addressed while expanding new nanostructured materials for their use in biosensors. Most nanobiosensor devices used in biomedical applications require a large sample for detection, which may lead to false-positive or false-negative results. Very few biosensors have attained commercial success at the global level, apart from electrochemical glucose sensors and lateral flow pregnancy tests. There is also a need for making nanostructure-based biosensors at an affordable cost that give rapid results with accuracy and are user-friendly. For example, nanomaterials should be integrated with a tiny biochip (lab-on-chip) for sample handling and analysis for multiplexed clinical diagnosis. More research should be done in this area and we expect the ongoing academic research to be realized into commercially viable prototypes by industries in near future.

## Figures and Tables

**Table 1:** Development of biosensors in different timelines.

Year	Development of Biosensor
1906	M. Cramer observed electric potential arising between parts of the fluid [20]
1909	Soren Sorensen developed the concept of pH and pH scale [21]
1909–1922	Griffin and Nelson were the first to demonstrate the immobilization of the enzyme invertase on aluminum hydroxide and charcoal [22,23]
1922	W.S. Hughes discovered a pH measurement electrode [24]
1956	Leland C. Clark, Jr invented the first oxygen electrode [8]
1962	Leland C. Clark, Jr et al. experimentally demonstrated an amperometric enzyme electrode for detecting glucose [9]
1967	Updike and Hicks and realized the first functional enzyme electrode based on glucose oxidase immobilized onto an oxygen sensor [10]
1969	Guilbault and Montalvo demonstrated and reported the first potentiometric enzyme electrode-based sensor for the detecting urea [11]
1970	Discovery of ion-sensitive field-effect transistor (ISFET) by Bergveld [25]
1973	Guilbault and Lubrano defined glucose and a lactate enzyme sensor based on hydrogen peroxide detection at a platinum electrode [12]
1974	K. Mosbach and B. Danielsson developed enzyme thermistor [13]
1975	D.W. Lubbers and N. Opitz demonstrated fiber-optic biosensor for carbon dioxide and oxygen detection [14]
1975	First commercial biosensor for glucose detection by YSI [26,27]
1975	Suzuki et al. First demonstrated microbe-based immunosensor [28]
1976	Clemens et al. demonstrated first bedside artificial pancreas [15]
1980	Peterson demonstrated the first fiber-optic pH sensor for in vivo blood gases [29]
1982	Fiber-optic biosensor for glucose detection by Schultz [30]
1983	Liedberg et al. observed surface plasmon resonance (SPR) immunosensor [19]
1983	Roederer and Bastiaans developed the first immunosensor based on piezoelectric detection [31]
1984	First mediated amperometric biosensor: ferrocene used with a glucose oxidase for glucose detection [18]
1990	SPR-based biosensor by Pharmacia Biacore [32]
1992	Handheld blood biosensor by i-STAT [32]
1999	Poncharal et al. demonstrated the first nanobiosensor [33]
2018	S. Girbi et al. demonstrated nerve-on-chip type biosensor for assessment of nerve impulse conduction [34]

**Table 2:** Represents the list of various nanomaterials employed in the development of biosensors.

Nanomaterial	Analyte	Transducer	Linear Range	Detection Limit	References
Au NBPs	Aflatoxin B1 (AFB1) Aflatoxin B1 (AFB1)	SPR Impedimetric	0.1–500 nM 0.1–25 nM	0.4 nM 0.1 nM	[48]
Au NPs	Uranyl	Electrochemical	2.4–480 µg L^−1^	0.3 µg L^−1^	[128]
Au NPs	Pb^2+^	Fluorescent	50 nm–4 µm	16.7 nm	[129]
Au/CdS QDs/TNTs	Cholesterol H_2_O_2_	Electrochemical Electrochemical	0.024–1.2 mM 18.73–355.87 µm	0.012 µM 0.06 µM	[196]
Au NPs	E.coli	Electrochemical	10–106 CFU mL^−1^	15 CFU mL^−1^	[197]
Au NP-MoS_2_-rGO	Carcinoembryonic antigen (CEA)	SAW	36.58 ng mL^−1^	0.084 ng mL^−1^	[198]
Au/rGO	miENA-122	Electrochemical	10 µm–10 pm	1.73 pM	[199]
Au NPs/TiO_2_	H_2_O_2_	Electrochemical	65–1600 µm	5 µm	[200]
Ag NPs	H_2_O_2_ Glucose Fe^2+^	Colorimetric	0.05–7.5 µm 1.5–3.0 µm 1–90 µm	0.032 µm 0.29 µm 0.54 µm	[135]
Ag/Pd NPs	Ractopamine Clenbuterol Salbutamol	Electrochemical	0.01–100 ng mL^−1^ 0.01–100 ng mL^−1^ 0.01–100 ng mL^−1^	1.52 pg mL^−1^ 1.44 pg mL^−1^ 1.38 pg mL^−1^	[201]
Ag@CQDs-rGO	Dopamine	Electrochemical	0.1–300 µm	0.59 nm	[202]
Ag NP-MWNT	Glucose	Electrochemical	0.025–1.0 mM	0.01 mM	[203]
Ag NPs	Mucin 1	Electro-chemiluminescence	1.135 fg mL^−1^–0.1135 ng mL^−1^	0.37 fg mL^−1^	[204]
Pt NPs	Adrenaline	Voltammetric	9.99 × 10^−1^–2.13 × 10^−4^ mol L^−1^	2.93 × 10^−4^ mol L^−1^	[205]
Pt NPs/RGO-CS-Fc	H_2_O_2_	Electrochemical	2.0 × 10^−8^ M–3.0 × 10^−8^ M	20 nm	[206]
Pt-Fe_3_O_4_@C	Sarcosine	Amperometric	0.5–60 µm	0.43 µm	[207]
Pt NFs/PANi	Urea	Cyclic Voltammetry	20 mM	10 µm	[208]
Pt@CeO_2_ NM	Dopamine	Electrochemical	2–180 nM	0.71 nM	[209]
Pd/Co-NCNT	Hydrazin	Electrochemical	0.05–406.045 µm	0.007 µm	[139]
Pd/CNF/[M3OA]^+^[NTF2]^−^	H_2_		1.00–35.0 nM	0.33 nM	[141]
Cu NPs/Rutin/MWCNTs/IL/Chit/GCE	H_2_O_2_	Cyclic Voltammetry	0.35–2500 µM	0.11 µm	[143]
Cu/rGO-BP	Glucose	Electrochemical	0.1–2 mM	11 µm	[144]
Cu_2_O@CeO_2_-Au	PSA	Amperometric	0.03 pg mL^−1^	0.0001–100.0 ng mL^−1^	[210]
Ni/Cu MOF	Glucose	FET	1 µM–20 mM	0.51 µM	[147]
NiO/PANINS	Glucose	Amperometric	1–3000 µM	0.06 µM	[211]
NiO@Au	Lactic acid	Electrochemical	100.0 µM–0.5 M	11.6 µM	[212]
Co_3_O_4_ NCs	Glutamate	Electrochemical chip	10–600 µM	10 µM	[150]
Co_3_O_4_-Au	miRNA-141	Photo-electricalchemical	1 pM–50 nM	0.2 pM	[152]
MnO-Mn_3_O_4_@rGO	H_2_O_2_	Impedimetric	0.004–17 mM	0.1 µM	[213]
MnO_2_ NFs	Salmonella	Impedimetric	3.0 × 10^1^–3.0 × 10^6^	19 CFU mL^−1^	[214]
Fe_2_O_3_/NiO/Mn_2_O_3_ NPs	Folic acid	Electrochemical	0.1 nM-0.01 mM	96.89 ± 4.85 pM	[215]
ZnO-rGO	Dopamine	Cyclic Voltammetric	0.1–1500 pM	8.75 ± 0.64 pM	[216]
ZnO NRs	Phosphate	FET	0.1 µM–7.0 mM	0.5 mM	[180]
ZnO NFs	Amyloid	Optical	2–20 µL	2.76 µg	[217]
Ca/Al-ZnO NPs	CO_2_	Semiconductor	0.25–5 RH%	200 ppm	[218]
Cr doped SnO_2_ NPs	Riboflavin	Voltammetric	0.2 × 10^−6^–1.0 × 10^−4^ M	107 nM	[219]
TiO_2_/APTES	glucose	Impedimetric	50–1000 µmol	24 µmol	[220]
TiO_2_ NTs	Asulam	photoelectrochemical	0.02–2.0 ng mL^−1^	4.1 pg mL^−1^	[221]
MoO_3_@RGO	Breast cancer	Electrochemical	0.001–500 ng mL^−1^	0.001 ng mL^−1^	[222]
Graphene QDs	Cu^2+^	Electrochemical	0.015–8.775 µM	1.34 nM	[168]
Graphene QDs	Lung cancer^+^	Fluorescence	0.1 pg mL^−1^–1000 ng mL^−1^	0.09 pg mL^−1^	[169]
CdTe/CdS//ZnS core/shell/shell QDs	l-ascorbic acid	Fluorescence	8.0 × 10^−9^–1.0 × 10^−7^ M	1.8 × 10^−9^ M	[170]
NSET amptamer@Fe_3_O_4_@GOD and MoS_2_	Tumor cell(EpCAM)	Magnetic fluorescence	2–64 nM	1.19 nM	[171]
Au NPs@PDA@CuInZnS QDs	P53 gene	Electrochemiluminescenece	0.1–15 nmol L^−1^	0.03 nmol L^−1^	[223]
CaM/SiNW-FETs	Protein	FET	10^−8^–10^−6^ M	7 nM	[224]
Si NWs	Dengue virus	FET	1 µM–10 fM	2.0 fM	[176]
ZnO NRs	Phosphate	FET	0.1 µM–7.0 mM	0.5 mM	[180]
G/Au NR/PT	HPV DNA	Electrochemical	1.0 × 10^−13^–1.0 × 10^−10^ m L^−1^	4.03 × 10^−14^ m L^−1^	[225]
Graphene-Au NRs	NADHEthanol	AmperometricVoltammetric	20–160 µM5–377 µM	6 µM1.5 µM	[226]
LAC-CNTs-SPCE	Para-cresol	Electrochemical	0.2–25 ppm	0.05 ppm	[227]
Co_3_O_4_-CNT/TiO_2_	Glucose	Photoelectrochemical	0–4 mM	0.16 µM	[228]
CNT thin-film transistor (TFT)	DNA	Thin film transistor (TFT)	1.6 × 10^−4^–5 µmol L^−1^	0.88 µg L^−1^	[229]
GQDs-MWCNTs	Dopamine	Electrochemical	0.005–100.0 µM	0.87 nM	[230]
CNT/Au NPs	Choline	Amperometric	0.05–0.8 mM	15 µM	[231]
PAMAM dendrimer	DENV 2E	Optical fiber	0.1 pM–1 µM	19.53 nm nM^−1^	[194]
SAM/NH_2_rGO/PAMAM	DENV 2E	SPR	0.08 pM–0.5 pM	0.08 pM	[195]
